# Supplementary figures and images for: Distribution and habitat of the painted tree rat (Callistomys pictus): Evaluating areas for future surveys and conservation efforts
Source: PLoS One. 2025 Jan 24;20(1):e0317356. doi: 10.1371/journal.pone.0317356 (PMC11759351; doi:10.1371/journal.pone.0317356)

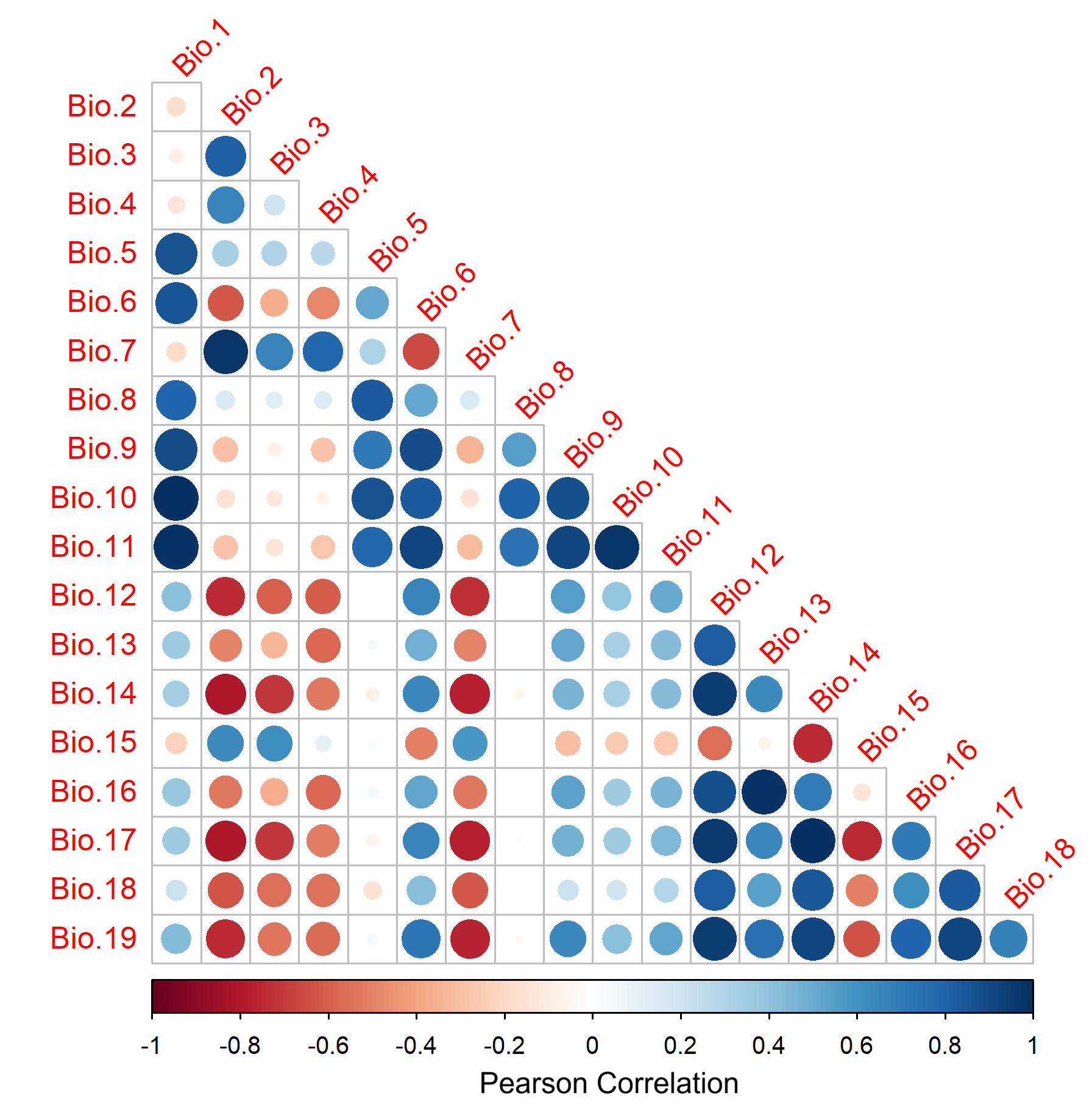

Supplement: S1 Fig — Variables with Pearson’s correlation coefficients below 0.70 were selected: Annual mean temperature (Bio1), isothermality (Bio3), temperature seasonality (Bio4), temperature annual range (Bio7), precipitation of the wettest month (Bio13), and precipitation seasonality (Bio15). (TIF) [file pone.0317356.s004.tif]

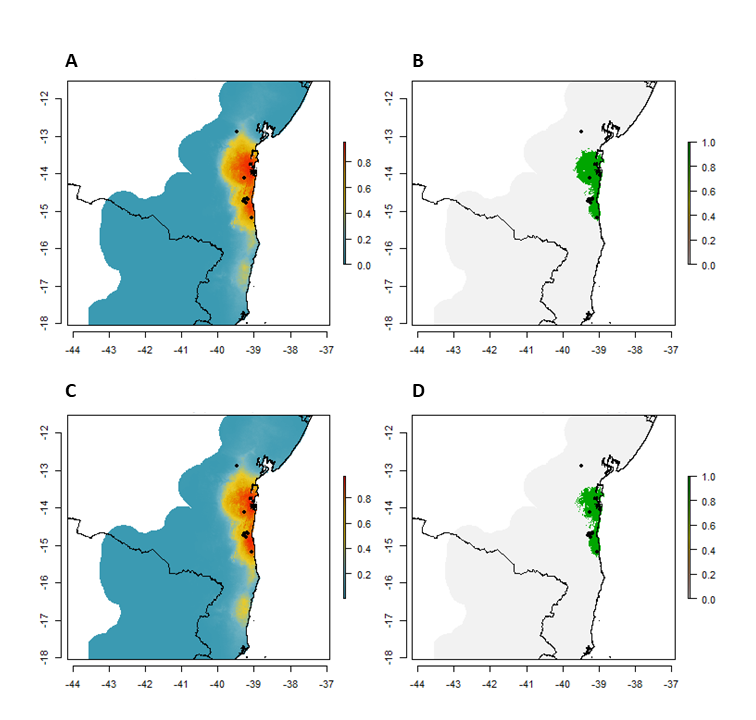

Supplement: S2 Fig — Comparison of the results from the single "best" model (A and B) and the ensemble model (C and D). The single "best" model is selected based on the lowest AIC value, while the ensemble model is based on the average values from the 10% of models with the lowest AIC values. A and C show the climate suitability gradient for the painted tree-rat (Callistomys pictus) as predicted by the best continuous model and the ensemble continuous model, respectively. B and D depict the core areas (highlighted in green) defined by the "10th percentile training presence" threshold. In the continuous models, the gradient from red to blue represents the shift from higher to lower climate suitability for the species. (TIF) [file pone.0317356.s005.tif]

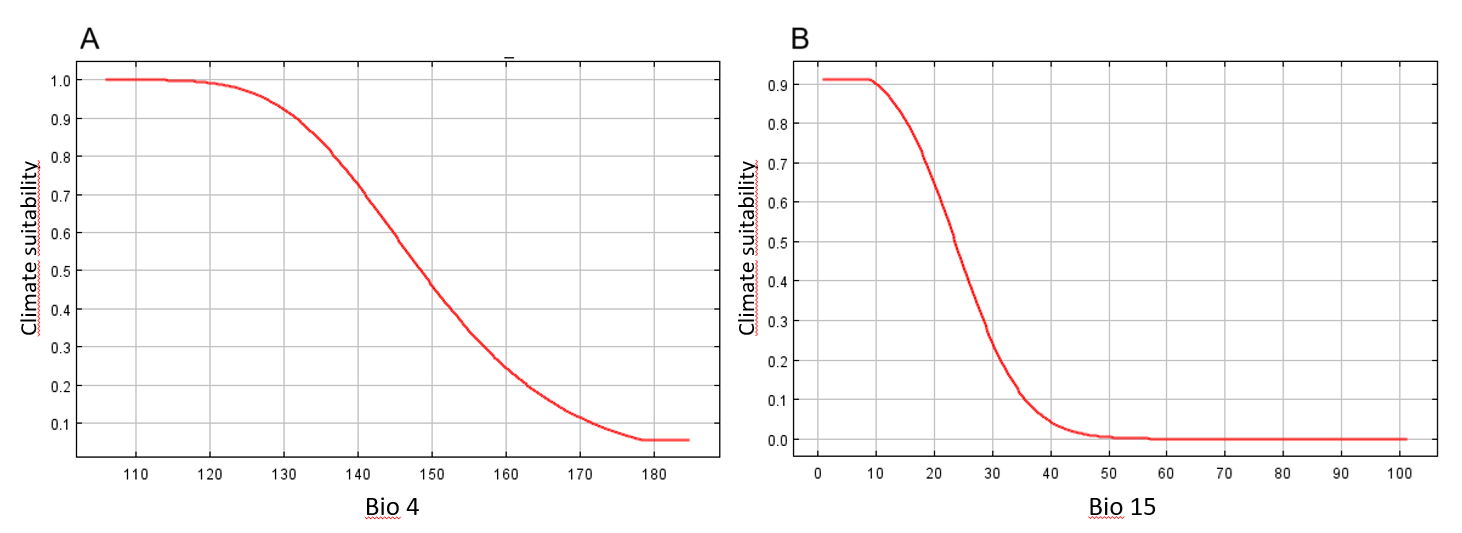

Supplement: S3 Fig — (TIF) [file pone.0317356.s006.tif]
