# Supplementary material for: Distribution and habitat of the painted tree rat (Callistomys pictus): Evaluating areas for future surveys and conservation efforts
Source: PLoS One. 2025 Jan 24;20(1):e0317356. doi: 10.1371/journal.pone.0317356 (PMC11759351; doi:10.1371/journal.pone.0317356)
Supplement: Attachment — Submitted filename: comments for the authors.docx [file pone.0317356.s007.docx]

Manuscript Number: PONE-D-24-33359

"Distribution and Habitat of the Painted Tree Rat (*Callistomys pictus*): Evaluating Areas

for Future Surveys and Conservation Efforts"

Comments to Author (also see the attached pdf file):

The manuscript deals with ecological niche modeling of current distribution of painted tree-rat (*Callistomys pictus*). Standard bioclimatic predictors from the WorldClim database and the species occurrence data according to the global biodiversity information and other published sources have been used to assess the potential distribution of the species using the maximum entropy modeling algorithm for the first time. Hence, the aim of the paper is rather ambitious.

Overall, the paper is concise and quiet well written, reasonably clear and the range of analytical methods are appropriate and well used. Statistical analysis is well described and conducted rigorously. However, I honestly think that basing such a study on a very small dataset with few point localities for the species can be dangerous. I suppose that less than at least 30 points cannot be representative of the distribution of a species at a national scale, nor trustable. Anyway, with regards to the author explanations throughout the text and the nature of the species, one can accept such an analysis for future surveys and conservation efforts of a rare unknown animal.

It should be considered that the presence-only data sets often suffer from spatiotemporal autocorrelation and therefore, potentially introduce environmental bias into modeling.
To address the issues related to the use of occurrence-only data in ecological modeling, I have the following suggestion which need authors’ attention: to correct sampling bias, it is important to spatially rarefy data depending on habitat and climatic heterogeneity to minimize environmental bias and to make it spatially independent. This is done with the rarefying tool available in the ArcGIS toolbox implemented in ArcMap and accounting sampling bias with a bias grid (I can refer authors to e.g., Brown, 2014. SDM toolbox: a python-based GIS toolkit for landscape genetic, biogeographic and species distribution model analyses. Methods in Ecology and Evolution, 5, 694–700. <https://doi.org/10.1111/2041-210X.12200>).

I would also suggest showing the bioclimatic variables used to develop the distribution model in Maxent as a new table, along with the contribution ratio (%) and permutation importance value for each layer. Moreover, the response curves of the variables with the greatest effect on the output of the model of current conditions can be added as the supplementary material.

I have also minor comments to do (also see the attached pdf file):

1. Please ensure that your manuscript meets PLOS ONE's style requirements.
2. I would like to recommend an English review since there are some small punctuation mistakes and not clear/long sentences, which sometimes are difficult to understand.
3. References seems to be a mixture of different formats and in somehow, not well addressed.

Finally, my recommendation for this manuscript, is “Accept after major revision”.
